# Thoracic Epidural and Intercostal Nerve Cryoablation in Multimodal Analgesia for Acute Pain Management of Rib Fractures: A Case Report

**DOI:** 10.7759/cureus.76187

**Published:** 2024-12-22

**Authors:** Nicholas Coccoluto, Chase Penhallurick, Vendhan Ramanujam

**Affiliations:** 1 Anesthesiology, Rhode Island Hospital, Brown University, Providence, USA

**Keywords:** acute pain services, regional anesthesiology, rib fractures, thoracic epidural analgesia, ultrasonography (usg)

## Abstract

Acute pain service was consulted for acute pain management in a 40-year-old male who had sustained multiple bilateral rib fractures following a fall injury. In addition to the rib fractures, the patient had also experienced injuries to his lungs and spinal column, both of which required surgeries. Considering the significant nature of pain due to his rib fractures, a multimodal pain management approach that included both pharmacological and non-pharmacological strategies was utilized. A thoracic epidural was performed by the acute pain service to provide immediate onset analgesia, especially during his stay in the hospital. At the same time, the surgeon performed thoracic intercostal nerve cryoablation, where nerves are frozen to prevent them from sending pain signals to the brain to provide analgesia for the patient even after getting discharged from the hospital. Both were done during a perioperative period. The cryoablations complemented the epidural, and they ensured continuous pain control in this patient. This report is unique as literature on utilizing both thoracic epidural and intercostal nerve ablations as part of a multimodal pain management approach for rib fractures is sparse.

## Introduction

Rib fractures are among the most common traumatic injuries seen in medicine, with evidence of the injury being reported in over 20% of thoracic trauma patients [1]. It causes significant patient morbidity, such as atelectasis, hypoxia, pneumonia, respiratory failure, poor pain control, and disability. Moreover, mortality rates can be as high as 30% in rib fracture patients [2]. For this reason, substantial efforts have been made to investigate various treatment regimens for rib fractures. Paramount to this is adequate acute pain management, which has been shown to reduce morbidity and mortality [3]. Recent efforts have emphasized multimodal analgesia through the combination of traditional pain medications and procedure-based interventions to achieve a synergistic effect. As an intervention, thoracic epidural is a commonly used technique that utilizes continuous infusion of local anesthetic medication into the epidural space surrounding the spinal cord to control pain and improve outcomes [4]. Other possible nerve blockade techniques include the injection of local anesthetics at spaces adjacent to the spinal column called the paravertebral block, surrounding the individual nerves coming out of the spinal nerves that supply the thorax called the intercostal nerve blocks, and into spaces between two layers of fascia surrounding muscles outside the spinal column where the branches of spinal nerves travel called as the thoracic fascial plane blocks; however, all their use is short-term and restricted to only when the patient is admitted to the hospital. For continued pain control even after the epidural is discontinued, freezing of the intercostal nerves, which is termed cryoablation, is a possible intervention. It has traditionally been used since the 1970s for pain control in thoracic surgeries [5]. Literature is sparse on using both epidural and intercostal nerve cryoablation together for acute pain control in rib fracture patients who do not receive surgical fixation of their fractures. We present a case of a patient with multiple rib fractures in whom we performed both thoracic epidural and intercostal nerve cryoablation to achieve effective and sustained analgesia.

This report was previously presented as a meeting abstract at the 49th Annual Regional Anesthesiology & Acute Pain Medicine Meeting on March 21, 2024. 

## Case presentation

As the case report lacks patient-identifiable information, it is exempt from institutional review board review requirements as per Lifespan policy. Nevertheless, patient informed consent was obtained for submission. This manuscript adheres to the case reports (CARE) guidelines.

**Figure 1:**
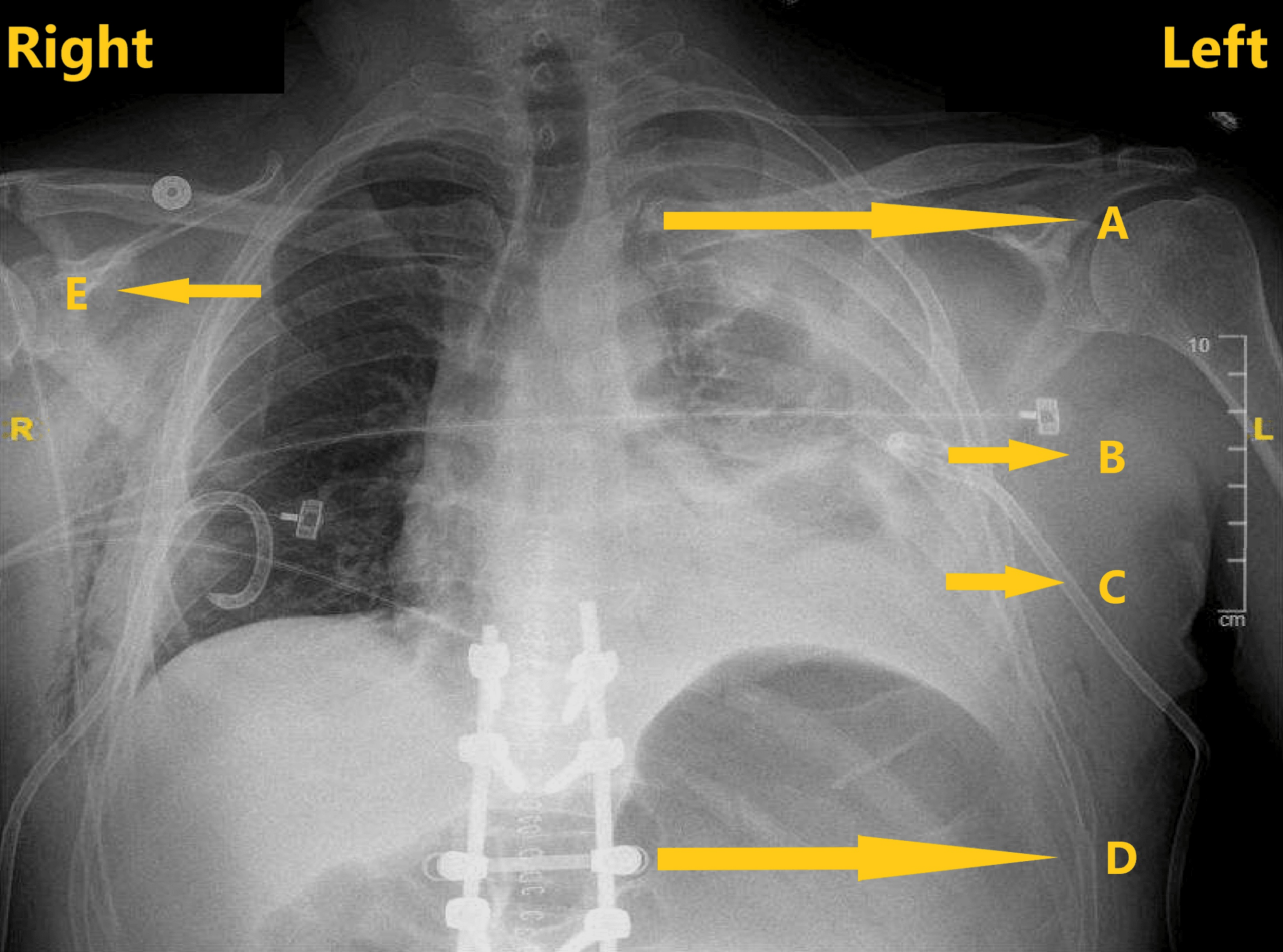
Chest radiography demonstrating rib fractures, pneumothorax, hemothorax, chest tubes and postsurgical hardware in spine A) rib fracture; B) chest tube; C) hemothorax; D) postsurgical hardware; E) pneumothorax

A 40-year-old male with a body mass index of 26.2 kg/m^2^ was admitted to the hospital following a fall after slipping from a truck. The patient sustained multiple rib fractures: left-sided displaced posterior fractures (second to eleventh ribs), left-sided non-displaced anterior fractures (eighth to eleventh ribs), and right-sided non-displaced anterior fractures (second and third ribs). He also had bilateral pneumothoraxes requiring chest tube placement, a left-sided hemothorax, and a non-displaced manubrium fracture. He had a thoracic level twelve vertebral burst and lumbar level three compression fractures that required urgent thoracic level ten to lumbar level three spinal fusion. On day two, after his admission and above-mentioned spine surgery, the acute pain service was consulted for his rib fracture pain management. On evaluation, regarding his rib fractures, he reported severe pain on the entire left side of his chest that was aggravated by any movement and relieved only with pain medications. His back pain following the spine surgery was moderate, with a pain score range between four to six on a numeric rating scale (NRS) and controlled with pain medications. He denied any other complaints. He denied a history of smoking, illicit substance use, or any known drug allergies. For pain, he was taking oral acetaminophen, gabapentin, oxycodone, intravenous ketorolac, and hydromorphone. Other medications included subcutaneous enoxaparin for deep vein thrombosis prophylaxis, oral methocarbamol for muscle relaxation, and oral senna-docusate for constipation. His physical examination revealed intermittent tachycardia with a heart rate above hundred beats per minute; oxygen saturation in mid-nineties while on three liters of supplemental oxygen through a nasal cannula; and blood pressure and temperature within normal limits. He had bilateral chest tubes and reduced bilateral breath sounds on the pulmonary examination. The rest of his systemic assessments were unremarkable. His laboratory tests were within normal limits except for blood hemoglobin of 9.3 g/dL, white blood cell count of 16.9 x 10^9^/L, and platelet count of 122 x 10^9^/L. His electrocardiogram was within normal limits. Radiography and computed tomography of the chest on both sides revealed demonstration rib fractures, small pneumothoraxes with reduced left hemothorax, chest tubes unchanged in position, and visualization of surgical changes between thoracic level ten and lumbar level three following the thoracolumbar fusion (Figure 1).

The same day, he was scheduled for a left-sided video-assisted thoracoscopic surgery for evacuation of residual and persistent hemothorax. His pain was poorly controlled and was significant on the left side of his chest due to the rib fractures and chest tube despite being on multimodal agents. Considering the risk for exacerbation after the surgery and the long duration of recovery of the rib fractures, left intercostal nerve cryoablations for long-term pain control were planned. To complement it, as well as for the pain arising from fractures and an indwelling chest tube on the right side of his chest, a thoracic epidural for short-term pain control was planned during the perioperative period. Since the right-sided rib fractures and their associated pain were not significant, right intercostal nerve cryoablations were not considered. During the surgery and under video-assisted thoracoscopic guidance, the surgeon performed the intercostal nerve cryoablations of the left second to eleventh nerves by placing the cryoablation probe (AtriCure®, Mason, Ohio, USA) under the lower edge of the posterior ribs with a temperature of -60 °C for two minutes for each nerve. Postoperatively, after obtaining clearance from the spine surgeon and verifying 12 hours of appropriate discontinuation duration of enoxaparin, the thoracic epidural catheter was placed by the acute pain service. With the patient in a sitting position, using the American Society of Anesthesiologists monitoring and sterile techniques, the epidural catheter was placed between thoracic levels six and seven using a loss of resistance technique [6]. A 17-gauge x 8.57-cm Tuohy needle and 19-gauge x 90-cm single open end hole catheter (Arrow®, FlexTip Plus®, Morrisville, USA) were utilized for this purpose. After a loss of resistance was attained at the 7 cm skin depth of the needle, the catheter was advanced. Its correct positioning in the epidural space was confirmed following a negative aspiration of the catheter for either blood or cerebrospinal fluid and a negative test dose administration of 3 mL of lidocaine-epinephrine (preservative-free) 1.5% - 1:200,000 in the catheter to rule out either intrathecal or intravascular catheter placement. It was then secured at 12 cm skin depth.

Within hours of epidural placement, the patient started experiencing significant improvement in pain control and respiratory status and was able to be weaned from supplemental oxygen. In the following days, he was able to demonstrate continued clinical improvements with a reduction in his opioid requirements, increased incentive spirometry efforts, and improved participation in physical therapy compared to before his interventions. The catheter was maintained for the next seven days with a continuous infusion of 0.1% bupivacaine at 10 mL per hour and as-needed patient-controlled intermittent boluses of 2 to 4 mL every 30 minutes. Since he was not able to be trialed off from the epidural due to his debilitating pain that was mostly from his manubrium fracture, considering the benefits of continuing the epidural for pain control and the risk of developing catheter infections, it was replaced between thoracic levels five and six using the same technique. The same epidural dosing was continued for another three days before discontinuation of the epidural when his pain had improved and become tolerable. But his opioid consumption, which kept gradually decreasing, increased during this three-day period, probably due to an increase in his physical therapy and activities. A consistent bilateral loss of sensations between thoracic dermatomes two and ten was achieved during the epidural use. During the ten days when the epidural was used, oral acetaminophen, as-needed intravenous hydromorphone, and oral oxycodone were administered as other pain medications. He was also switched from subcutaneous enoxaparin to heparin for deep vein thrombosis prophylaxis. The patient was subsequently discharged home forty-eight hours later with as-needed oral oxycodone for pain management. There were no complications related to these interventions and no other significant events during the hospital stay. During the following three months, the patient was able to be titrated down on his oxycodone use, which he reported to be taking only for pain related to his back surgery. His rib fractures had healed, and he was able to complete his physical therapy and return to his daily routine. He reported some degree of sensory loss along the distribution of the ablated nerves on the left side of his chest and stated that it did not affect his functionality.

Table 1 presents the patient's pain-related data collected during his management and follow-up. Reported mean pain scores were calculated from an even number of pain scores reported by the patient over a twenty-four-hour period using a numeric rating scale (NRS) pain scale. Total opioid consumption over that twenty-four-hour period is reported in milligrams as morphine milligram equivalents (MME). The mean volume of inspiratory breath taken by the patient while using incentive spirometry is reported in milliliters. As reported earlier, two days after the patient's admission, the first epidural and cryoablation were performed. The first epidural was replaced after seven days of use and the replaced second epidural was used for the next three days before discontinuation. He was finally discharged two days later and then followed up after three months. 

**Table 1:** Patients pain related data A - Day before the first epidural and cryoablation; B - Day after the first epidural and cryoablation; C - Day seven after the first epidural and cryoablation; D - Day three after the replacement of the first epidural; E - Three months after discharge from hospital; NRS - numeric rating scale; MME - morphine milligram equivalents

	A	B	C	D	E
Chest mean NRS pain score	7.8	7	7.4	6	2
Opioid consumption in MME as milligrams	131.25	112.5	13.75	90	7.5
Mean volume of inspiratory breaths during incentive spirometry in milliliters	1250	1500	2000	2500	2500
Activities	Bed exercises	Out-of-bed exercises	Ambulation	Self-care	Daily routine

## Discussion

Pain is the most common symptom following rib fractures, and its early aggressive management is crucial to mitigate both short- and long-term complications. The use of multimodal analgesia regimens has been advocated for pain management in rib fractures due to their improved analgesia and reduction in opioid intake and side effects [5, 7]. Multimodal analgesia relies on synergistic combinations of medications to decrease dosing requirements and minimize adverse drug reactions for any single medication [7]. For rib fractures, the multimodal analgesia approach includes the use of opioids and non-opioids such as non-steroidal anti-inflammatory drugs, acetaminophen, skeletal muscle relaxants, alpha-2 agonists, N-methyl-D-aspartate (NMDA) receptor antagonists, neuropathic pain medications such as gabapentin, topical analgesics, and interventional therapies. In the setting of rib fractures, a multimodal approach has been suggested to address pain control and respiratory performance [8].

As an interventional therapy, thoracic epidural analgesia is a popular intervention. It is achieved by placing a catheter in the thoracic epidural space and anesthetizing the spinal nerves entering and leaving the spinal cord through local anesthetics administered via the catheter. The local anesthetics bind to the voltage-gated sodium channels in the nerves in their open or inactivated state and prevent sodium ions from entering the neurons. This blocks the generation and propagation of action potentials and thereby prevents the nerve conduction of electrical impulses. It has demonstrated improved pain control and outcomes, including a lower frequency of pneumonia, shorter ventilator duration, shorter hospital stays, and reduced mortality in rib fracture patients [8-10]. Although there are other alternatives such as paravertebral, erector spinae, and intercostal nerve blocks, these interventions have inferior effectiveness as they lead to the blockade of only one-sided nerves compared to thoracic epidural where blockade of multiple spinal nerves arising from the spinal cord on both sides of the chest happen, and they are used as interventions only when an epidural is either challenging to accomplish, not indicated or contraindicated [11, 12]. The opioid consumption following these blocks has been reported to be significantly higher than the epidural starting from recovery to up to twenty-four hours [11]. While thoracic epidural has beneficial outcomes, there are risks associated with the procedure, such as the development of an epidural hematoma or abscess; hemodynamic instability such as hypotension and bradycardia; medication overdosing or toxicity; analgesia failure due to catheter dislodgment; and leakage or occlusion when they are used for a long period [13]. Hence, their use is restricted to short-term and only to patients admitted to the hospital where they can be closely monitored. These risks can be mitigated by monitoring the patient adequately during all parts of the day and changing the epidural catheter after use of up to seven days, as was done in this case. Following spine surgery, there can be concerns for infections, hematoma, and interference with neurological monitoring when epidural analgesia is administered. However, previous studies have shown that there is no significant difference in the side effects while using epidural analgesia following major spine surgeries and that there is significantly superior analgesia, higher patient satisfaction, and decreased overall opioid consumption [14]. This was the scenario in this case, especially when the spine surgeons gave clearance for the epidural, and the benefits of epidural analgesia were considered significant for patient recovery.

Rib fractures take several weeks to heal, and patients who suffer from bilateral and multiple rib fractures can continue to experience pain after getting discharged. This can lead to chronic pain and disability. Consequently, there is a need for pain control regimens even after discontinuation of an epidural and patient discharge from the hospital. Besides pharmacological agents, intercostal nerve cryoablation (cryo-neurolysis) can be helpful. Cryoablation of nerves is the direct application of cold temperature (approximately up to −70 °C) using a probe on the nerves for a few minutes, resulting in reversible Wallerian degeneration of those nerves and post-degeneration analgesia that can last for six to twelve months [15]. The effect of cryoablation depends on the temperature applied and the degree of nerve degeneration that happens. It can be done either thoracoscopically in the operating room or percutaneously at the bedside, with both demonstrating comparable efficacy [16]. Complication rates are low and include neuroma development, prolonged sensory dysfunction along the ablated nerves, and injury to nearby structures [16]. The incidence of post-ablation neuralgic pain development, which was a concern in the past, is currently found to be low [17]. Although intercostal nerve cryoablation has been there for a while, recently, it has gained popularity for its use in conjunction with surgical stabilization of rib fractures for refractory chest wall symptoms where conventional therapies have failed. This combination has been demonstrated to lower pain scores and opioid consumption and reduce the length of hospital and intensive care unit stays [5, 16, 18]. With surgical fixation of rib fractures not commonly performed in all rib fracture patients, the use of intercostal nerve cryoablation without surgical fixation for rib fracture pain control is limited. They have been reported to facilitate extubation and improve incentive spirometry use [19]. In our case, in combination with epidural and other multimodal analgesia agents, intercostal nerve cryoablation may have contributed to pain control and recovery. Since cryoablation takes several days to take effect, epidural and other analgesics will be necessary for pain management to begin with, and by the time the epidural is discontinued after several days of use, the effects of cryoablation can take over to continue uninterrupted analgesia that can provide progressive pain control with reduced opioid intake and improved functionality for the patient. This is evident from the decrease in opioid consumption in this patient, which reduced from before intervention 131.75 mg per day to 13.75 mg per day on the seventh day after the intervention, then to 7.5 mg per day at three months after the intervention. During the same periods of time, his incentive spirometry efforts improved, and he progressed from bed exercises to self-care and finally returned to his daily routine level of activities. Pain management in rib fracture patients following a trauma can be complex as they can be frequently encountered with other injuries that can contribute to the overall pain and recovery, as seen in this case where the spine injury and manubrium fracture contributed to a rise in an occasional opioid consumption to 90 mg per day from 131.75 mg per day on the 10th day after the intervention and extended the duration of epidural use until 10 days. The urgent need for spine surgery to stabilize the spine was the only reason for both interventions being performed the day after the patient's admission following his trauma. 

This report is not without limitations. Being a case report of a single patient, the findings are retrospective, non-comparable to other therapeutic interventions, and might be over-interpreted. It cannot be generalized to a broader population. The intercostal nerve cryoablations and their implications described here cannot be generalized and validated as the patient had multiple sources of pain, such as underlying spine injury that required fixation, surgery for left chest hemothorax, and some degree from opposite side chest injury that did not receive cryoablation.

## Conclusions

In conclusion, intercostal nerve cryoablation can be considered as a possible intervention along with thoracic epidural and other multimodal analgesia agents for multiple rib fracture pain control. However, this is only a case report of a single patient with multiple injuries that makes understanding of the effects of intercostal nerve cryoablation undetermined and non-generalizable. Chest Wall Injury Society has included intercostal nerve cryoablation as a priority research interest in chest wall injury patients as the existing evidence is limited. Hence, future studies such as randomized control trials evaluating intercostal nerve cryoablation alone and intercostal nerve cryoablation in combination with thoracic epidural for multiple rib fracture pain control following trauma and other related outcomes will help address the knowledge gap on their impact on rib fracture pain management.
